# Validation of Digital Impressions’ Accuracy Obtained Using Intraoral and Extraoral Scanners: A Systematic Review

**DOI:** 10.3390/jcm12185833

**Published:** 2023-09-08

**Authors:** Naisargi Shah, Mrinmyaee Thakur, Shruti Gill, Omkar Shetty, Nasser M. Alqahtani, Mohammed A. Al-Qarni, Saeed M. Alqahtani, Mohamed Fadul A. Elagib, Saurabh Chaturvedi

**Affiliations:** 1 Department of Prosthodontics, Terna Dental College, Navi Mumbai 400706, Maharashtra, India; drnaisargi69@gmail.com (N.S.); dr.mrinmayeet@gmail.com (M.T.); shrutz.287@gmail.com (S.G.); 2 Department of Prosthodontics, SGT Dental College Gurugram, Gurgaon 122505, Haryana, India; oshetty123@gmail.com; 3 Department of Prosthetic Dentistry, College of Dentistry, King Khalid University, Abha 62529, Saudi Arabia; nmalqahtani@kku.edu.sa (N.M.A.); smaalqahtani@kku.edu.sa (S.M.A.); 4 Department of Restorative Dentistry, College of Dentistry, King Khalid University, P.O. Box 3263, Abha 61471, Saudi Arabia; maalqarny@kku.edu.sa; 5 Division of Periodontics, Department of Periodontics and Community Dental Sciences (PCS), College of Dentistry, King Khalid University, Abha 62529, Saudi Arabia; mfdel@kku.edu.sa

**Keywords:** intraoral scanners, extraoral scanners, accuracy, trueness, precision

## Abstract

Background: At present, the evidence regarding digital impressions’ accuracy recorded by using digital scanners is lacking. This systematic review aimed to evaluate whether the type of scanning (intraoral/extraoral) affects the Accuracy of Digital Impressions. Method: Two independent reviewers performed a systematic search in the database both electronically and manually (PubMed, Ebsco HOST, the Cochrane Library, and Google Scholar) for articles published from 1 January 2010 to 1 December 2022. This study was registered with the International Prospective Register of Systematic Reviews (PROSPERO CRD42020188765) and followed the PRISMA statement. The question in focus was as follows: Does the type of scanning (intraoral or extraoral) affect the accuracy of digital impression? Results: A total of 449 papers were obtained by searching electronically and manually. In total, 15 complete-text papers qualified for assessment based on eligibility criteria. After reading the full-text articles, five studies were excluded. Ten studies were selected for the qualitative analysis. The qualitative data reported that the accuracy of both types of scanners (intraoral and extraoral) lies within the range of clinical acceptability. Nevertheless, the intraoral scanners seem to be more accurate when compared to the extraoral scanners for a partial arch situation. Conclusions: Scanning type affects the accuracy of the digital impression. Various factors influence the scanning ability. Intraoral scanners seem to be more accurate compared to extraoral scanners for a partial arch situation. More studies comparing the accuracy of the intraoral scanner and extraoral scanner for a complete arch scan and in an in vivo study setting are needed.

## 1. Introduction

A dental impression is a routine procedure used to record oral tissues. The casts fabricated are used for diagnostic purposes, treatment planning, or restoration fabrication. The impression should accurately represent the oral tissues of the patient, and imprecise surface details may influence the adaption, fit and eventually longevity of the restoration [1,2,3]. Conventional impression has been the standard of practice for many decades [1]. Few limitations, such as dimensional changes in dental stones and the volumetric changes of impression materials, lead to inaccuracies. Digital scanners and CAD-CAM (computer-aided design and manufacturing) technologies were created for precise, dependable impressions to overcome these challenges with conventional approaches [4]. 

The first step of the CAD-CAM system, data collection, includes either intraoral or extraoral scanners. There are two methods to record impressions digitally: the direct approach and the indirect approach [5,6]. The direct approach includes intraoral scanner systems, which enable the acquisition of data from intraoral tissues. The indirect approach includes either impression scanning or model scanning. These techniques record the intraoral tissues of a patient and transform them into 3D data that can be digitally saved and further altered with CAD software [7,8,9,10,11,12,13]. They provide easier treatment planning, case acceptance, laboratory communication, reduced operative time and storage requirements [4].

A CAD-CAM-derived prosthesis has to undergo multiple steps like data collection, data processing and manufacturing that can cumulatively affect the fit of the prosthesis [10,14,15,16,17,18,19]. There are two possibilities to analyse the accuracy of data acquired from intraoral and extraoral scanners. Firstly, to compare the fit of the restoration or to compare the surface tessellation language (STL) datasets with a reference dataset [20].

The parameters of accuracy are “Trueness” and “Precision”. ISO (International Organization for Standardization) Norms 5725 and 12836 were applied for the accuracy of digital models [21,22]. Trueness is the degree to which the arithmetic means of many test findings closely resemble the true or widely accepted reference value [21]. While precision measures how closely test results agree with one another [22].

The accuracy of intraoral scans is affected by complex environmental factors, such as patient movement [23,24], saliva [23,24], limited space [23], presence of blood and gingival crevicular fluid [24], the translucency of the material being scanned [2], light conditions [25] the reflection of light off the tooth surface [26], the experience of the operator [27], and humidity [23]. The extraoral scan accuracy is affected by the dimensional inaccuracies of the impression material, dental stone and operator error.

Advancement in image acquisition is an ongoing evolution. With the advancement in digital scan technologies, these may end up outperforming traditional impression methods [5,28,29,30]. Moreover, numerous scientific articles have resulted from the increased interest in the use of digital technologies. There are articles where tooth-supported crowns fabricated using digital and conventional impression techniques present similar marginal discrepancies [28]. However, there is ambiguity regarding the accuracy of the digital impression obtained from the digital scanner [23,25]. Hence, this systematic review was performed with the aim to evaluate the current status of digital scanners (intraoral or extraoral) on the accuracy of digital impressions. Additionally, not all studies not have both parameters of accuracy but are still included to evaluate the current status of digital scanners.

## 2. Materials and Methods

The presented systematic review was performed following the PRISMA (Preferred reporting items for systematic reviews and meta-analysis) guidelines 2009 [31]. This review was registered in PROSPERO with the registration number CRD42020188765.

### 2.1. Research Strategy

The PICO-focused [32] question was “Does the type of Scanning (Intraoral or Extraoral) affect the Accuracy of Digital Impression?” The P-Population was documented as digital impressions, which can be recorded by using various digital scanners. I-Intervention was considered digital impressions captured from intraoral scanners and C-Comparison was related to digitalized impressions captured from extraoral scanners. O-Outcome measure was the accuracy of a digital impression in terms of trueness or precision.

### 2.2. Eligibility Criteria

Inclusion criteria were studies on digital impressions of dentulous patients or models. Since this systematic review is aimed at comparing types of scanning, patients or model with or without preparation were considered. Studies measuring Accuracy, trueness, or precision, studies including any aspect of software or techniques related to intraoral and extraoral, and studies using any one or multiple scanning systems with a minimum of five scans. All the relevant literature published in the English language from 1 January 2010 to 1 December 2022 was included.

Exclusion criteria were studies involving complete edentulous arch scans, studies on complete conventional impressions, and studies considering comparison or relationship between digital and traditional impression methods. Additionally, studies include impressions in implants, magnetic resonance imaging (MRI) scans, and in-process trials. Studies where extraoral scan impression was recorded using material other than elastomers were excluded.

### 2.3. Data Extraction

The search for the studies, which were published from 1 January 2010 to 1 December 2022, was performed using electronic means and manual methods. The electronic databases included PubMed, EBSCOhost, The Cochrane Library, and Google Scholar. The search was conducted using controlled vocabulary terms, Medical Subject Heading (MeSH terms) like the Dental Impression technique, Data measurement accuracy and Data accuracy, and free-text terms. Keywords used for systematic searches were digital impression, intraoral scanner, extraoral scanner, digital scanners, accuracy, trueness and precision. Boolean operators (AND and OR) were used to combine the terms. Phrase searching, truncation and wild cards were used wherever necessary (Table 1). A similar search strategy was implemented in all electronic searches. Dental journals were manually searched for topics on digital dentistry and digital prosthodontics in the institution’s library.

### 2.4. Quality Assessment

Two reviewers independently conducted electronic and manual searches. In total, 447 papers were obtained via electronic means and 2 papers were collected by searching manually, thus making a total of 449 articles. All the articles were exported into the Mendeley Desktop 1.19.6 software. The ‘check for duplicates’ feature of this software was then used to identify and eliminate duplicates. Each article that was detected as a duplicate by the software was checked meticulously.

**Figure 1:**
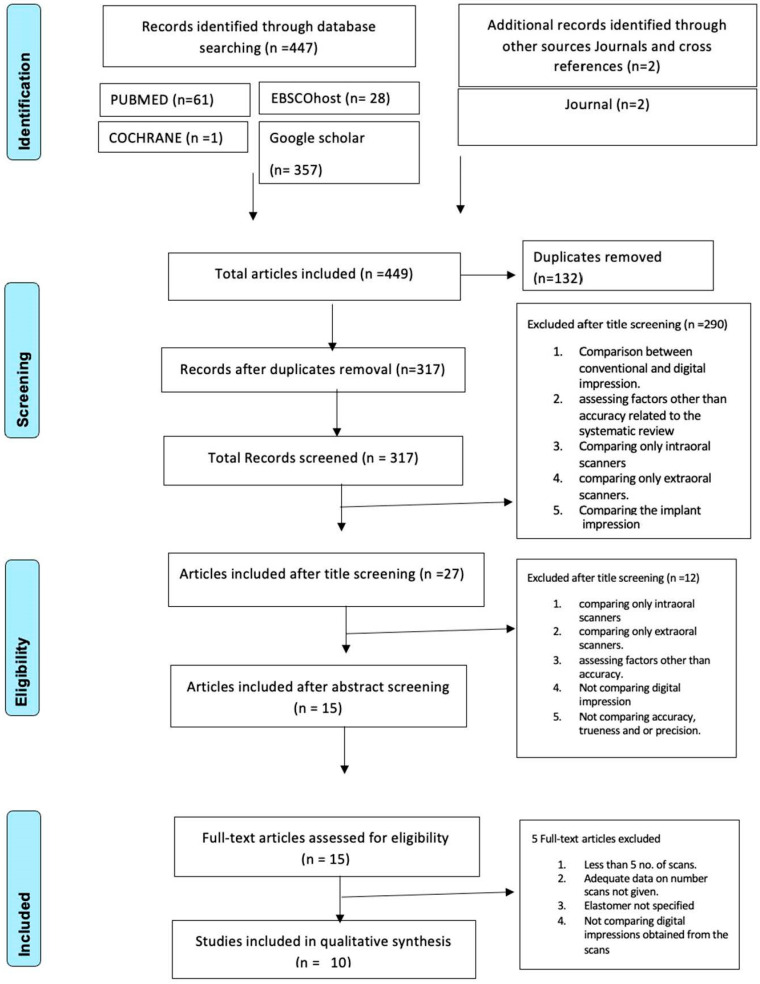
Shows the method of data selection (PRISMA 2009 Flow Diagram).

For the collection of the systematic data, three steps procedure was followed. Step-1 assessment and screening of the data by two independent reviewers. In this step, reviewers screened the titles of the papers gathered electronically or via a manual method of searching; then, step 2 involved the screening of the abstracts. The complete texts of the papers were next examined by the two reviewers in step 3. The study was included for additional screening in the event of doubt or disagreement between the reviewers. The final decision was then taken by the third reviewer. The PRISMA 2009 Flow Diagram presented in Figure 1 shows the method of data selection [33]. By using Cohen’s kappa, Inter-reviewer reliability was evaluated [34]. The K values were found for Google Scholar (K = 0.85), Cochrane (K = 1), Ebsco Host (K = 0.83), and PubMed (K = 0.84).

## 3. Results

### 3.1. Studies and Quality Selection

The evaluation of the database resulted in the identification of 449 papers. After the assessment of the titles of the papers and their abstracts, 15 complete text papers were chosen for assessment under the eligibility criteria. After reading the full-text papers, five studies were excluded (Table 2). Ten studies (three in vivo and seven in vitro studies) were included in this systematic review [3,5,20,23,26,35,36,37,38,39]. The data were subsequently extracted from the included studies and recorded in the data extraction sheet (Table 3).

It was observed that the accuracy of the extraoral scanners was in the acceptable range. Additionally, accurate digital impressions obtained from intraoral scanners are better or similar than those from the extraoral scanner.

Since the selected studies had heterogeneous data like different reference datasets, reference scanners, points of measurement, test samples (full arch scans and single tooth scan), and inspecting software, this systematic review was restricted to a qualitative analysis.

### 3.2. Risk of Bias

Two independent reviewers used the revised and validated version of the Methodological items for non-randomized studies (MINORS) scale to assess the risk of bias in the included studies [43,44]. The MINORS scale was adapted to conform to the requirements of the included studies (Table 4). The risk of bias for individual studies was calculated. For each study, the summary of the bias risk for the 12 points was plotted (Table 5). The scores were classified as follows

- Low risk of bias (bias unlikely to materially affect the outcomes);
- Unclear risk of bias (a bias that casts some doubt on the findings);
- High risk of bias (a bias that significantly reduces credibility regarding the findings in the paper).

**Figure 2:**
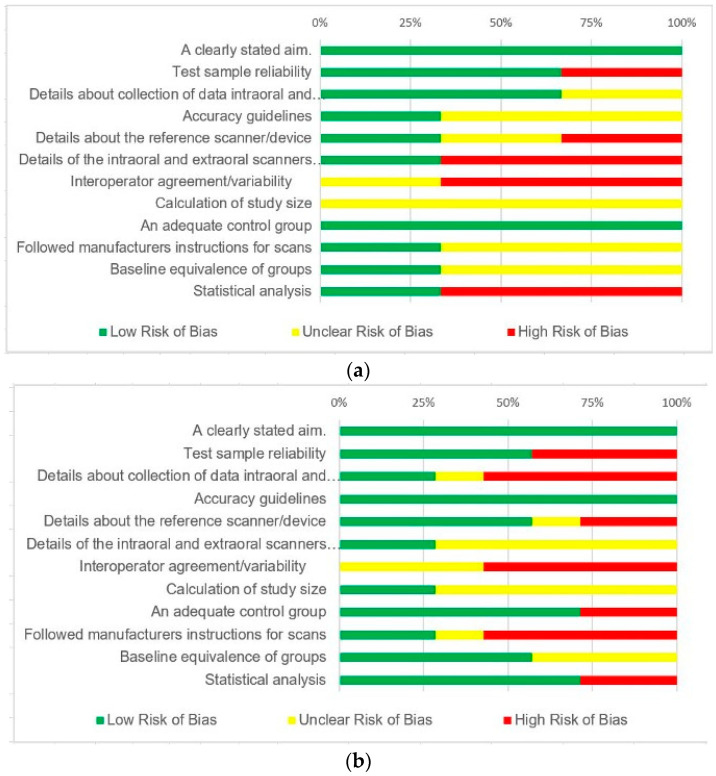
(**a**) Risk of bias (in vivo studies); (**b**) risk of bias (in vitro studies).

The studies included displayed an overall low risk of bias, as demonstrated in Table 5, indicating a good quality of evidence. The evaluated results show that the included studies were generally of excellent quality and a high risk of bias was only evident in certain specific observations (Figure 2a,b).

The precision of digital impressions was evaluated by Flügge et al. [23]. Bohner et al. [39] determined the trueness and the accuracy was evaluated by Vecsei et al. [35], Sason et al. [26] and Muallah et al. [38]. Some studies [35,38] evaluated the combined accuracy of digital impressions. Flügge et al. [23], Kuel et al. [3] and Sason et al. [26] conducted clinical studies. Complete arch scans were conducted by Flügge et al. [23]^,^ and Muallah et al. [38]. Most studies evaluated partial and single tooth scans [3,5,20,35,36,37,39]. Vescei et al. [35], Sason et al. [26] and Muallah et al. [38] evaluated and analysed the accuracy of the linear measurements between the landmarks on the model. By superimposing the scans, Flügge et al. [23] and Bohner et al. [39] compared the precision and trueness of the impression. Except for Flügge et al. [23], every other study concluded that intraoral scanners provide more accurate digital impressions than extraoral scanners.

## 4. Discussion

The role of digital scanners is promising as they provide better accuracy, efficiency and comfort [45,46,47]. The scanner (either extraoral or intraoral) is utilized to transfer the data of the oral cavity into computer software. Indirect digitization has been in use for quite some time. Extraoral scans are performed by scanning gypsum casts and conventional impressions [2,5,20,23]. Even with the proven accuracy, they depend on impressions or casts, thus limiting the benefit of digital technology. On the other hand, the intraoral scanner performs a direct scan of the oral cavity. It is comfortable, and efficient, and provides virtual casts that can be easily stored and communicated with the laboratory [24]. Despite the promising advantages, there are limitations associated with the present-day intraoral scanners, which calls upon their comparison with extraoral scanners and conventional impressions. Digital impressions are promising alternatives to conventional impressions [24]. However, the role of the intraoral scanner is yet to be established.

In this systematic review, the studies included evaluated multiple factors influencing the accuracy of the scanner like the extent of the area, surface physical properties of the object (reflective, matte and porous) [23,39], technologies (triangulation, confocal microscopy) [39] and the inspecting software. The factors impacting the accuracy of digital impressions are discussed below.

For full-arch scans, higher precision for complete-arch scans was observed in an in vivo study using an extraoral scanner compared to an intraoral scanner. For partial arch scans, however, the intraoral scanner provided more accurate scans when compared to the full arch within clinically acceptable accuracy [3,35,37,38].

In intraoral scanning, image acquisition is incrementally defined. Therefore, additional scans of complex angled surfaces are obtained from different angles than the flat axial surface, leading to bloated image data. Additionally, in the scanning of such extensive and complex angled areas, there is a merging of multiple single images, which causes progressive distortion due to the stitching of the multiple images. The software uses the first image made by the scanner as a reference and all subsequent images are stitched to the previous one. Each overlap has an inherent error which would gradually increase with every stitching process. It causes higher inaccuracy of the resulting dataset, leading to a systematic error. Hence, the longer the scanning field, the longer the stitching processes and the larger the errors would be [26]. However, the extraoral scanner continuously captures the projection of laser planes and the recording of their reflections from all directions (simultaneously) [23]. So, the area or the complex surface are not the factors influencing the extraoral scanner. Thus, in the case of ios (Intra-Oral Scanners), shorter directly measured distances showed decreased deviations, whereas longer directly measured distances showed higher deviations.

Digital impressions obtained using a digital scanner are affected by the surface physical properties of the object as different materials detect and reflect light differently [3,26,39].

Subsurface scattering (SSS) is a phenomenon where the light that has been scattered inside of an object is emitted at a different position and angle than it would have been if it had reflected off the surface directly. The accuracy of the digital impression is decreased because SSS light affects the measurements of directly reflected surface light detected by using an intraoral scanner sensor [25].

To overcome this, there are scanning devices that require the titanium dioxide coating in powder form, which acts as an opaque reflector on the teeth before scanning. They eliminate reflection to create a proportionate surface for increasing scan accuracy [48]. The matte finish prevents reflections during image capture, which improves the detection of the preparation like a finish line. Powder application limits their practicability and accuracy because it adds to the measuring error due to the thickness of the coating powder [37,38]. Moreover, it creates different microscopic planes, which cause the incident rays to scatter, resulting in a diffuse reflection [26]. It was reported that powder leads to errors of up to 40 μm [25,39]. A poor quality of the coating results in inaccurate scans. Errors resulting from improper powder coating have lost their relevance as today’s advanced IOS scanners operate without powder [25]. The most modern scanners are optical 3D video scanner systems that can produce multiple 3D video data images per second and can record data in real time and true colour without the need for powder application [48,49].

Scanning technologies also affect digital impressions. Bohner et al. [39] compared digital scanners with confocal laser and triangulation scanning technologies. In triangulation, three beams of light are directed and focused together on a similar point. If there is a surface which presents an uneven dispersion of light, then the accuracy may get hampered as noted in most cases. Confocal laser scanning microscopy acquires high-resolution optical in-focus images from selected depths point-by-point. The laser is used as a source of light for scanning intraorally and to achieve accurate results. The laser in the form of the light source and confocal laser scanning microscopy (CLSM) technique together facilitates the capture of enormous image data and transfer into the computer. It helps in reconstructing the profile surface of an opaque sample and also helps in obtaining the interior imaging of a non-opaque sample [50,51]. However, there was no significant difference between these two technologies [39].

Apart from the technologies used for the scanner, different inspecting software is used for comparing the datasets. The majority of research superimposes test datasets with precise reference datasets using best-fit techniques. The digital models’ surfaces’ point-to-point spatial differences are measured. The approach of best fit appears to be appropriate for the short arch (one quadrant), as the error resulting from the superimposition itself between the test and reference datasets is within a reasonable range [5]. However, the superimposition method increases the influence of the inaccuracy with larger datasets. Therefore, a highly accurate CMM (coordinate measuring machine) measurement of a geometrical item was employed in order to circumvent the best-fit algorithm’s constraint. It allows for the accurate measurement of angular and linear shifts in all three dimensions [5].

The accuracy of intraoral scanners is also influenced by scanning protocol, soft tissue span, the number of additional scans, and the automatic correction of missing data [35]. Scanning processes have an impact on the accuracy of digital impressions captured using digital scanners. Passive and active strategies are clearly separated in the realm of 3D reconstruction. Passive approaches, which depend on an object’s texture to some extent, illuminate intraoral tissues only via ambient light. Active approaches, which rely less on the actual texture and colour of tissues for reconstruction, use white, red, or blue structured lights projected from the camera onto the object [4]. The movements of the scanner during various scanning procedures have also received extensive study. According to the order of surfaces examined, the six techniques studied by Faris Z. Jamjoom et al. [52] were labelled as follows. B-O-P: Beginning posteriorly, moving along the ridge’s buccal aspect to the other side, returning along the occlusal aspect, and lastly scanning the palatal or lingual aspect. The P-O-B method involves starting posteriorly, moving along the ridge’s palatal or lingual aspect, turning around and moving along the occlusal aspect, and then scanning the buccal aspect. O-B-P: Starting posteriorly, moving along the ridge’s buccal aspect back to the opposite side, and lastly scanning the palatal or lingual aspect. Beginning posteriorly, moving along the ridge’s occlusal aspect to the opposite side, returning along the palatal or lingual aspect, and lastly scanning the buccal aspect. ZZ-P: Starting posteriorly on the occlusal aspect of the ridge, moving zigzag-style down the ridge between the occlusal and buccal aspects before scanning the palatal or lingual aspect. ZZ: For the mandible, start posteriorly on the buccal aspect of the ridge, move to the opposite side crossing the palate, continue in a zig-zag path scanning the entire ridge, and end anteriorly. For the maxillary arch, start posteriorly on the buccal aspect, move to the opposite side crossing the palate, continue in a zig-zag path scanning the entire arch, and end anteriorly. They discovered that the P-O-B strategy had the highest overall accuracy [52]. However, no scanning type or technology can be considered more precise and accurate because of the lack of standardized procedures or study settings. With the available literature, the intraoral scans are comparable to extraoral scans in partial arch situations. However, full arch scans are still a challenge due to the above-cited reasons. The study by Sason GK et al. [26] showed that average precision values ranged from 20.7 to 33.35 for IOS scanner and from 19.5 to 37 for the extraoral scanner. They concluded that the intraoral scanner showed higher “precision” and “trueness” values when compared with the extraoral scanner. Patzelt et al. [53,54] in their study found the trueness of four IO scanners ranging from 44.1 to 591.8 μm for edentulous arches and 38 to 332.9 completely edentulous arches, respectively, and the authors concluded that except for one intraoral scanner, all other tested systems showed comparable levels of trueness values. These studies suggest that scanning with IOS in partially edentulous arch provide more accurate results. Various studies evaluated the IOS and extraoral scanner and it had been elucidated that significant differences in the trueness and precision between the different intraoral and laboratory scanners exist. However, in most studies, the difference was clinically acceptable, the trueness values ranged from 7.7 to 46 μm, and precision values ranged from 4 to 50 μm., for the extraoral scanner [55,56,57].

Risk of bias was performed using a revised and validated version of the Methodological items for the non-randomized studies (MINORS) scale. A high risk of bias was associated owing to inadequate information on the scanners’ details, inspecting software, scanning protocol, and personal experience.

The selected studies presented heterogeneous data right from the technology of the scanner used, the extent of the area being scanned (full arch scans and single tooth scan), reference scanners, points of measurement or methods used to compare the accuracy and inspecting software. There is growing interest in the utilization of digital scanners and also scientific research related to them. However, due to the continuous upgradation of software and new technologies, it is difficult to have similar inspecting parameters. The heterogeneity of the impression technique, the material, and the system introduces an inevitable form of inaccuracy. In addition, studies have considerable variation in terms of evaluation methods, inspecting software, and operating principles. This reflects the conflicting outcomes of the studies whose results are still inconclusive. More studies evaluating the accuracy of the digital impression with similar methodologies are needed. It could help the dentist delineate the proper techniques with minimal error, thereby enhancing their performance quality. The use of scanners intraorally has definite limitations in the form of anatomical structures and involves soft and hard tissues, the presence of saliva, insufficient light, and, above all, patient cooperation. The errors would be more pronounced in the posterior area than in the anterior. Furthermore, the sequence scanning and the process of stitching the recorded images may affect the accuracy in posterior areas. Scanning and stitching can accumulate variance in the scans. This variance would be more pronounced in the mandibular arch than in the maxillary arch, due to obvious anatomical constrictions as tongue. Thus, more in vivo studies are needed to understand the related ramifications of clinical conditions over the controlled environment setting, as well as in vitro studies.

Hence, it is necessary to establish a universally accepted and standardized method for digital impression accuracy evaluation.

**Limitations**

- Most of the studies have not individually specified the trueness and precision of scans but have instead combined the accuracy of scans.
- With an ongoing advancement in scanning technologies, it is difficult to have a standardized format to assess the scanners’ inaccuracies.

## 5. Conclusions

Within the limitations of this systematic review, the following conclusions are drawn:

- The accuracy of digital impressions recorded using the digital scanners is affected by the scanning techniques.
- The intraoral scanner seems more accurate compared to extraoral scanners under partial arch scans. However, the accuracy shown by extraoral scanners is also within the clinically acceptable range.
- Studies comparing the accuracy of a scanner (intraoral and extraoral) in an in vivo setting are lacking for complete arch scans.
- Further studies with similar methodology (PICO question—Does the type of scanning (intraoral or extraoral) affect the accuracy of digital impression?) with strict inclusion criteria of clinical studies in all available electronic databases, specifically assessing the role and effect of clinical parameters during the use of digital scanners both intraoral and extraoral, are needed to validate the accuracy of digital scanners under clinical conditions.

## Figures and Tables

**Table 1:** Pico search strategy.

**POPULATION**	(((((Dental Impression Technique [MeSH Terms]) OR (Dental Impression Technique * [Tiab])) OR (Optical digital impression * [Tiab])) OR (Dental digital impression * [Tiab])) OR (Digital Impression * [Tiab])) OR (Virtual impression * [Tiab])
**INTERVENTION**	((((((((((((Intraoral scanner * [Tiab]) OR (Intraoral scanning * [Tiab])) OR (Intraoral scanning system * [Tiab])) OR (Intraoral digital scanner * [Tiab])) OR (Direct digital impression * [Tiab])) OR (Intraoral digital impression * [Tiab])) OR (Intraoral digitization * [Tiab])) OR (direct intraoral digitization * [Tiab])) OR (Intraoral acquisition * [Tiab])) OR (Direct digital scan * [Tiab])) OR (Optical scanner * [Tiab])) OR (Dental laser scanner * [Tiab])) OR (Dental light scanner * [Tiab])
**COMPARISION**	(((((((((((Extraoral scanner * [Tiab]) OR (Extraoral scanning * [Tiab])) OR (Extraoral scanning system * [Tiab])) OR (Indirect digital impression * [Tiab])) OR (Extraoral digital impression * [Tiab])) OR (Extraoral digitization * [Tiab])) OR (extraoral digital scanner * [Tiab])) OR (Extraoral acquisition * [Tiab])) OR (Indirect digital scan * [Tiab])) OR (Optical scanner * [Tiab])) OR (Dental laser scanner * [Tiab])) OR (Dental light scanner * [Tiab])
**OUTCOME**	((((((((((Dimensional Measurement Accuracy[MeSH Terms]) OR (Dimensional Measurement Accuracy * [Tiab])) OR (Data Accuracy[MeSH Terms])) OR (Data Accuracy * [Tiab])) OR (Accuracy * [Tiab])) OR (accuracies * [Tiab])) OR (Trueness * [Tiab])) OR (Exactness * [Tiab])) OR (Correctness * [Tiab])) OR (Precision * [Tiab])) OR (Repeatability * [Tiab])

**Table 2:** List of excluded studies.

Study ID	Author	Title	Reason for Exclusion
1	Su et al. [14]	Comparison of the repeatability between intraoral digital scanner and extraoral digital scanner: an in vitro study	The material used for the extraoral scan was not mentioned.
2	Rudolph et al. [30]	Accuracy of intraoral and extraoral digital data acquisition for dental restorations	An inadequate number of Intraoral Scans.
3	Shimizu et al. [40]	The Accuracy of the CAD system using intraoral and extraoral scanners for designing of fixed dental prostheses	Number of Scans not mentioned.
4	Wesemann et al. [41]	Accuracy and efficiency of full arch digitalization and 3D printing: A comparison between desktop model scanner, an intraoral scanner, a CBCT model scan, and stereolithographic 3D printing	Comparison of digital impressions not available.
5	Pagano et al. [42]	Evaluation of the Accuracy of Four Digital Methods by Linear and Volumetric Analysis of Dental Impressions	An inadequate number of scans.

**Table 3:** Data extraction sheet of included studies.

Study Characteristics																			
												Mean				Standard Deviation			
				Number of scans								Intraoral scanner		Extraoral scanner		Intraoral scanner		Extraoral scanner	
Id	Author	Year	Study Type	Intraoral scans	Extraoral scans	Impression material	Reference data	Test sample	method for comparing accuracy	Evaluated		Trueness	Precision	Trueness	Precision	Trueness	Precision	Trueness	Precision
1	Flügge TV et al. [23]	2013	in vivo	20	20	polyether	1st scan	full arch			Precision		50 (mean deviation)		25				
					20										10				
2	Sason at al. [26]	2018	in vivo	30	30	addition silicone	prepared tooth measured intraorally with digital vernier calliper	distance between the dimples on teeth		Trueness	Precision	MD: 477 BL: 349	20.7 to 33.35	MD: 456 BL: 336	19.5 to 37	MD: 576.4 BL: 655	MD: 19.6 BL: 16.4	MD: 743 BL: 626.5	MD: 24 BL: 22.5
3	Keul C and Guth JF [3]	2019	in vivo	12	24	polyether	metal bar data set made using coordinate measuring instrument	metal bar		Trueness	Precision	Vectorial error: M-SCAN: 287.4		Vectorial error: M-IMP: 318 M-CAST: 256		Vectorial error: M-SCAN: 88.4		Vectorial error: M-IMP: 150 M-CAST: 159	
												Angulation: M-SCAN: 0.46		Angulation: M-IMP: 0.38 M-CAST: 0.42		Angulation: M-SCAN: 0.11		Angulation: M-IMP: 0.13 M-CAST: 0.36	
				12	24							Vectorial error: P-SCAN: 305.1		P-IMP: 412.8 P-CAST: 517		P-SCAN: 157.1		P-IMP: 185.3 P-CAST: 627	
												Angulation: P-SCAN: 0.40		Angulation: P-IMP: 0.82 P-CAST: 0.91		Angulation: P-SCAN: 0.14		Angulation: P-IMP: 0.45 P-CAST: 1.27	
4	Guth et al. [20]	2013	in vitro	12	12	polyether	A CAD/CAM-fabricated titanium model of prepared teeth scanned with industrial ct	prepared teeth on titanium model		Trueness	Precision	1.5		3.6		5.2		1.9	
5	Guth et al. [5]	2015	in vitro	12	12	polyether	metal bar attached to polymeric full arch model and measured with cmm	metal bar		Trueness	Precision	8.9		7.7		4.8		3.6	
6	Vecsei et al. [35]	2016	in vitro	30	10	polyvinyl siloxane	pmma maxillary cast scanned with ref scanner	prepared teeth on pmma master cast		Trueness	Precision	sd: 22.31		sd: −40.26		sd: 40		sd: 79.67	
												md: 115.82		md: 5.18		md: 50.67		md: 111.32	
												ld: −163.45		ld: −325.81		ld: 145.47		ld: 134.13	
7	Guth et al. [36]	2016	in vitro	12	12	polyether	A CAD/CAM-fabricated titanium model of prepared teeth scanned with industrial ct	prepared teeth on titanium model		Trueness	Precision	1. CS 3500: 1.4 (1.4 to −1.3)		D-810 1.9 (2 to −1.8)		1. CS 3500 1 (1 to 1)		6. D-810: 6 (8 to 7)	
				12								2. Zfx Intrascan: 3.3 (3.7 to −2.9)				2. Zfx Intrascan 1.6 (1.5 to 1.9)			
				12								3. CEREC Bluecam: 2.9 (3.1 to −2.3)				3. CEREC Bluecam 3 (3 to 3)			
				12								4. CEREC Omnicam: 3.1 (3.0 to −3.2)				4. CEREC Omnicam 3 (4 to 2)			
				12								5. True Definition: 1.1 (1.2 to −1.0)				5. True Definition 2 (1 to 3)			
8	Lee et al. [43]	2016	in vitro	6	6	polyvinyl siloxane	pmma model scanned with enginner scanner	prepared teeth on pmma master cast		Trueness	Precision	Bluecam: 17.5	Bluecam: 12.7	CS1: 17.4	CS1: 9.2	Bluecam: 1.8	Bluecam: 2.6	CS1: 1.7	CS1: 1.2
				6	6							Omnicam: 13.8	Omnicam: 12.5	CS2: 12.3	CS2: 6.9	Omnicam: 1.4	Omnicam: 3.7	CS2: 0.1	CS2: 2.6
9	Muallah et al. [38]	2017	in vitro	37∗6=	37	polyvinyl siloxane	A resin master model was created by 3D printing and mesured by coordinate measuring instrument	full arch		Trueness	Precision					**IMD**: 1. apollo di: 57.669		IMD: OrthoXscan: 34.006	
																2. CS3500: 99.76			
																3. iTero: 84.137			
																4. Plan Scan: 214.756			
																5. Trios: 52.872			
																6. True Definition: 169.298			
																**ICW**: 1. apollo di: 36.007		ICW: 37.206	
																2. CS3500: 43.39			
																3. iTero: 22.008			
																4. Plan Scan: 80.761			
																5. Trios: 22.351			
																6. True Definition: 42.347			
																**AL**: 1. apollo di: 64.859		AL: 57.27	
																2. CS3500: 84.442			
																3. iTero: 30.673			
																4. Plan Scan: 91.89			
																5. Trios: 23.205			
																6. True Definition: 62.065			
10	Bohner et al. [39]	2017	in vitro	10	10	polyvinyl siloxane	typhodont with acrylic teeth scanned with industrial ct	prepared teeth		Trueness		Cervical Region: 32.8		Cervical Region: 46.7		Cervical Region: 21.4		Cervical Region: 56.9	
												Axial Surface: 14.1		Axial Surface: 18.9		Axial Surface: 5.3		Axial Surface: 12.7	
												Occlusal Surface: 65		Occlusal Surface: 71.7		Occlusal Surface: 10.9		Occlusal Surface: 10.3	
				10	10							Cervical Region: 34.4		Cervical Region: 32.2		Cervical Region: 16.7		Cervical Region: 16.4	
												Axial Surface: 25.6		Axial Surface: 16.7		Axial Surface: 17.4		Axial Surface: 12.2	
												Occlusal Surface: 40.6		Occlusal Surface: 77.8		Occlusal Surface: 22.4		Occlusal Surface: 88.6	

MD: Mesio distal; BL: Bucco lingual; sd: Short Distance;md: Medium Distance; ld: Long Distance; CS1: Cast Scanner 1; CS2: Cast Scanner 2; IMD: Inter Molar Width; ICW: Inter Canine Width; AL: Arch Length.

**Table 4:** Minors tool modified as per systematic review.

	Minors	Minors Tool Adapted as per Systematic Review
1	A clearly stated aim	A clearly stated aim
2	Inclusion of consecutive patients	Test sample reliability
3	Prospective collection of data	Details about the collection of data intraoral and extraoral scans
4	Endpoints appropriate to the aim of the study	Accuracy guidelines considered
5	Unbiased assessment of the study endpoint	Details about the reference scanner/device
6	Follow up period appropriate to the aim of the study	Details of the intraoral and extraoral scanners
7	Loss to follow up less than 5%	Interoperator agreement/variability
8	Prospective calculation of the study size	Calculation of study size
9	An adequate control group	An adequate control group
10	Contemporary groups	Followed manufacturer’s instructions for scans
11	Baseline equivalence of groups	Baseline equivalence of groups
12	Adequate statistical analyses	Statistical analysis

**Table 5:** Summary of risk of bias of included studies.

Sr. No.	Minors Tool Adapted as per Systematic Review	Flügge, T.V. et al. (2013) [23]	Sason, G.K. et al. (2018) [26]	Keul, Güth, J.F. (2019) [3]	Güth, J.F. et al. (2013) [20]	Güth, J.F. et al. (2015) [5]	Vecsei, B. et al. (2016) [35]	Güth, J.F. et al. (2016) [36]	Lee, J.J. et al. (2016) [37]	Muallah, J. et al. (2017) [38]	Bohner et al. (2017) [39]
		In Vivo Studies			In Vitro Studies						
1	A clearly stated aim.	2	2	2	2	2	2	2	2	2	2
2	Test sample reliability	2	1	2	1	2	2	1	1	2	2
3	Details about collection of data intraoral and extraoral scans.	2	1	2	0	1	1	2	1	2	1
4	Accuracy guidelines	0	0	2	2	2	2	2	2	2	2
5	Details about the reference scanner/device	0	1	2	2	0	2	1	2	2	1
6	Details of the intraoral and extraoral scanners mentioned	1	1	2	1	1	1	2	1	1	2
7	Interoperator agreement/variability	1	1	0	0	0	1	1	1	1	0
8	Calculation of study size	0	0	0	0	0	0	0	0	2	2
9	An adequate control group	2	2	2	2	2	2	1	2	1	2
10	Followed manufacturers instructions for scans	2	0	0	0	1	2	1	2	1	1
11	Baseline equivalence of groups	0	0	2	0	2	2	2	2	0	0
12	Statistical analysis	1	2	1	1	2	1	2	2	2	2
	Total Score (Out of 24):	13	11	17	11	15	18	17	18	18	18

0 = Not reported; 1 = Reported but inadequate; 2 = Reported and adequate. Grading: Low risk: Score >/= 16; High risk: Score < 16.

## Data Availability

Data can be provided for academic purposes on request from the primary author.
